# 2408. Is Shorter Antibiotic Treatment Feasible in Patients with Cardiac Implantable Electronic Devices (CIED) Infections? Impact of Treatment Duration on 6-Month Recurrence and Mortality

**DOI:** 10.1093/ofid/ofad500.2028

**Published:** 2023-11-27

**Authors:** Giacomo Ponta, Martina Ranzenigo, Alessandra Marzi, Chiara Oltolini, Chiara Tassan Din, Caterina Uberti-Foppa, Vincenzo Spagnuolo, Patrizio Mazzone, Paolo Della Bella, Paolo Scarpellini, Antonella Castagna, Marco Ripa

**Affiliations:** San Raffaele University Hospital, Milan, Lombardia, Italy; San Raffaele University Hospital, Milan, Lombardia, Italy; San Raffaele University Hospital, Milan, Lombardia, Italy; Unit of Infectious Diseases, IRCCS San Raffaele Scientific Institute, Milan, Lombardia, Italy; San Raffaele University Hospital, Milan, Lombardia, Italy; San Raffaele University Hospital, Milan, Lombardia, Italy; Vita-Salute San Raffaele University; Unit of Infectious Diseases, IRCCS, San Raffaele Scientific Institute, Milan, Emilia-Romagna, Italy; San Raffaele University Hospital, Milan, Lombardia, Italy; San Raffaele University Hospital, Milan, Lombardia, Italy; San Raffaele University Hospital, Milan, Lombardia, Italy; IRCCS San Raffaele Hospital and Vita-Salute San Raffaele University, Milano, Lombardia, Italy; San Raffaele University Hospital, Milan, Lombardia, Italy

## Abstract

**Background:**

Aim of this study is to compare the 6-month outcomes (recurrence and mortality) of patients with cardiac implantable electronic device (CIED) infections treated with short (SHORT, <14 days) or long (LONG, >14 days) duration of antimicrobial therapy after device removal.

**Methods:**

Retrospective cohort study of patients hospitalized at IRCCS San Raffaele Hospital (Milan, Italy) from June 2011 to June 2021, who underwent device removal for CIED infection. Patients with pocket and lead infections were included, while valve endocarditis and *Staphylococcus aureus* infection were excluded.

The characteristics of patients in the SHORT and LONG groups were compared using the chi-square test for categorical variables and the Mann-Whitney U-test for continuous variables. A propensity score (PS) was calculated using a logistic regression that considered duration of therapy as the dependent variable; the association between patients’ characteristics and outcomes was evaluated with logistic regression analysis.

**Results:**

The characteristics of the 150 included patients are described in table 1, the microbial etiology in table 2. Patients in the SHORT compared to the LONG group were older (75 [65-80] vs 69 [62-77], p-value=0.047), had a higher Charlson comorbidity index (5 [3-6] vs 4 [3-5], p-value=0.021), had less frequent isolation of coagulase-negative staphylococci (CoNS; 46.7% vs 68.9%, p-value=0.006), and an unknown etiology (36.7% vs 13.3%, p-value=0.001). Moreover, the duration of antimicrobial treatment after device removal was shorter (13 [9-14] vs 20 [16-29] days, p-value< 0.001). At 6 months, recurrence and mortality were not significantly different between the SHORT and the LONG group (recurrences: 2 [3.7%] vs 5 [6.7%], p-value=0.464; mortality: 2 [3.7%] vs 3 [4.0%], p-value=0.931),

At logistic regression analysis (table 3 and table 4) adjusted for PS, treatment duration was not significantly associated with either recurrence (odds ratio [OR] 0.524; 95% confidence interval [95%CI] 0.084-3.263) or mortality (OR 1.402; 95%CI 0.185-10.635).

**Figure:**
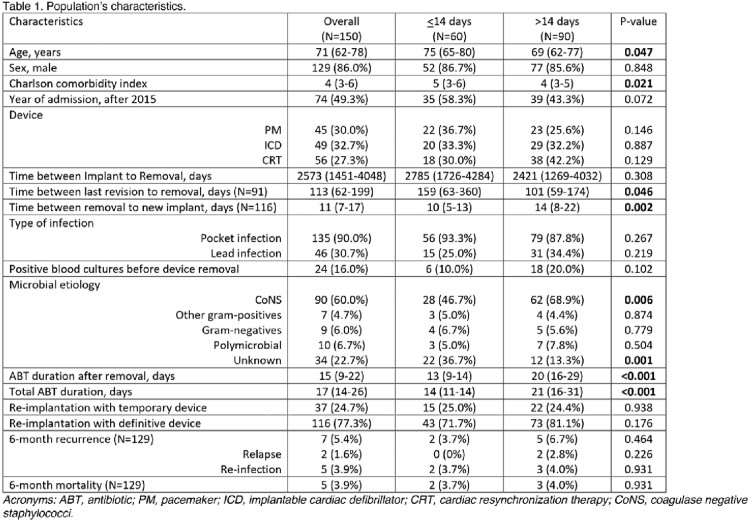

**Figure:**
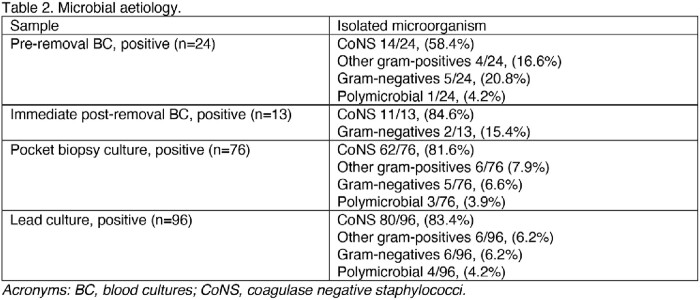

**Figure:**
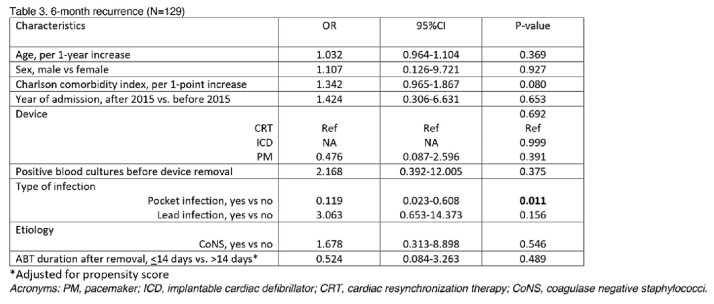

**Conclusion:**

A shorter duration of antimicrobial treatment after device removal may be an effective approach for treating patients with CIED infections.

**Figure:**
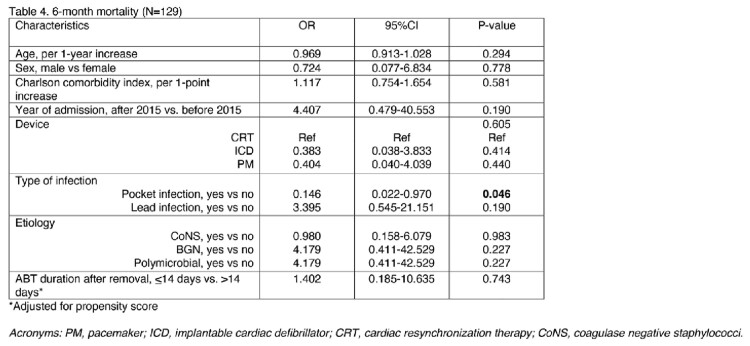

**Disclosures:**

**Antonella Castagna, MD**, Bristol-Myers Squibb: consultancy payments and speaking fees|Gilead: consultancy payments and speaking fees|Janssen-Cilag: consultancy payments and speaking fees|Merck Sharp & Dohme: consultancy payments and speaking fees|ViiV Healthcare: consultancy payments and speaking fees

